# Evaluation of Incompatibility Group I1 (IncI1) Plasmid-Containing *Salmonella enterica* and Assessment of the Plasmids in Bacteriocin Production and Biofilm Development

**DOI:** 10.3389/fvets.2019.00298

**Published:** 2019-09-06

**Authors:** Pravin R. Kaldhone, Ashlyn Carlton, Nesreen Aljahdali, Bijay K. Khajanchi, Yasser M. Sanad, Jing Han, Joanna Deck, Steven C. Ricke, Steven L. Foley

**Affiliations:** ^1^Division of Microbiology, U.S. Food and Drug Administration, National Center for Toxicological Research, Jefferson, AR, United States; ^2^Center for Food Safety and Food Science Department, University of Arkansas, Fayetteville, AR, United States; ^3^Department of Agriculture, University of Arkansas at Pine Bluff, Pine Bluff, AR, United States; ^4^Department of Biological Sciences, King Abdul-Aziz University, Jeddah, Saudi Arabia; ^5^Veterinary Research Division, Department of Parasitology and Animal Diseases, National Research Centre, Giza, Egypt

**Keywords:** *Salmonella enterica*, IncI1 plasmids, biofilms, bacteriocins, virulence, pMLST

## Abstract

Mobile genetic elements, such as plasmids, can potentially increase the ability of bacteria to infect and persist in vertebrate host cells. IncI1 plasmids are widely distributed in *Salmonella* from food animal sources and associated with clinically important strains. These plasmids often encode antimicrobial resistance; however, little is known about their impact on the virulence of *Salmonella* strains. To assess the potential impact of the plasmids on virulence, 43 IncI1-positive *Salmonella* isolates from human and animal sources were subjected to whole genome sequence (WGS) analyses and evaluated for their abilities to invade and persist for 48 h in Caco-2 human intestinal epithelial cells, form biofilms and encode bacteriocins. Draft WGS data were submitted to predict the presence of virulence and antimicrobial resistance genes, plasmid replicon types present, conduct plasmid multilocus sequence typing (pMLST), and core genome MLST (cgMLST) in the isolates. Caco-2 cells were infected with *Salmonella* strains and incubated for both one and 48 h for the invasion and persistence assays, respectively. Additionally, *Salmonella* isolates and IncI1 plasmid carrying transconjugants (*n* = 12) generated in *Escherichia coli* were assessed for their ability to produce biofilms and bacteriocin inhibition of growth of other bacteria. All *Salmonella* isolates infected Caco-2 cells and persisted in the cells at 48 hrs. Persistent cell counts were observed to be significantly higher than invasion assay cell counts in 26% of the isolates. Among the IncI1 plasmids, there were 18 pMLST types. Nearly 35% (*n* = 15) of *Salmonella* isolates produced biofilms; however, none of the IncI1-positive transconjugants produced increased biofilms compared to the recipient. Approximately 65% (*n* = 28) of isolates and 67% (*n* = 8) of IncI1-positive transconjugants were able to inhibit growth of at least one *E. coli* strain; however, none inhibited the growth of strains from species other than *E. coli*. The study characterized IncI1 positive *Salmonella* isolates and provided evidence about the potential contributions of IncI1 plasmids virulence phenotypes and areas where they do not. These findings should allow for more focused efforts to assess the impact of plasmids on bacterial pathophysiology and human health.

## Introduction

*Salmonella enterica* is estimated to cause more than one million infections resulting in 400 deaths per year in the U.S. (1). Most *Salmonella* infections are self-limiting and resolve with symptomatic treatment (2). In some cases, such as co-infection with other bacteria, infection of an immunocompromised host and/or infection by highly virulent strains of *Salmonella* can lead to more severe, invasive and sometimes fatal infections (3). In such cases, patients typically need antimicrobial treatment to recover from disease. The annual economic impact of all *Salmonella* infections has been estimated to be up to 4.4 billion dollars due to the cost of treatment, loss of wages, and quality of life (4). Most *Salmonella* infections are enteric in nature and foodborne salmonellosis is an important economic and public health concern (5, 6). The origin of foodborne salmonellosis can be eggs, poultry products, meat, and fresh produce (5–10). *Salmonella* is relatively widespread among poultry and other food animals (11, 12).

*Salmonella* maintains its high presence in diverse hosts through genetic plasticity (13), which allows *Salmonella* to alter its genetic composition to adapt to changing environmental conditions. This plasticity can be achieved with the help of mobile genetic elements such as plasmids. Plasmids that have been characterized in *Salmonella* can carry genes associated with increased antimicrobial resistance and virulence for their hosts (5, 14, 15). Plasmid transfer between bacteria leads to the potential for rapid horizontal spread of genes among bacteria. If these genes influence phenotypic characteristics, such as antimicrobial resistance and virulence, then a better understanding of genetic determinants could be vital to the management of foodborne illnesses arising from *Salmonella* (5).

Plasmids can be grouped based on their incompatibility to co-exist in the same cell (16). Plasmid incompatibility assays for typing are based on phenomena that prevent coexistence of plasmids with the same replication and division mechanisms in a bacterium (17). Incompatibility group I1 (IncI1) plasmids are commonly found in enteric bacteria from food animal sources and are associated with clinically relevant strains. They are known for their potential to carry and disseminate antimicrobial resistance genes among enteric pathogens (18, 19). For example, many of the plasmids associated with the dissemination of genes encoding resistance to ceftriaxone, an antimicrobial agent used in management of severe *Salmonella* infections, have been reported to be IncI1 plasmids (20).

Similarly, the spread of virulence-associated genes via plasmids could lead to *Salmonella-*related illnesses difficult to manage, which raises a potential public health concern. Bacterial virulence genes encode factors/molecules that aid a bacterium in colonization of host niche; entry into, survival within and exit from the host cell; evasion or suppression of the host immune response to the bacterium; and/or obtaining limited nutrients from the host environment (21, 22). These factors can include biofilm formation that aid in niche colonization, bacteriocin production that can limit niche competition, nutrient acquisition, secretion systems that facilitate bacterial uptake into host cells and improve intracellular survival, and mechanisms that regulate the expression of virulence mechanisms (22). Genes potentially associated with virulence have been identified on IncI1 plasmids (23); however very few, if any studies have been conducted to directly evaluate IncI1 plasmids and their virulence potential in *Salmonella*. Thus, the objectives of this study were to characterize *Salmonella* strains that harbor IncI1 plasmids, both for the presence of antimicrobial resistance and virulence genes and the potential to invade and persist in human intestinal epithelial cells; and to specifically assess the impact of IncI1 plasmids on the ability to produce biofilms and encode bacteriocins that could provide a selective colonization advantage for the strains that carry them.

## Materials and Methods

### Bacterial Isolates

Forty-three *S. enterica* isolates carrying IncI1 plasmids were selected for this study from a larger set of previously characterized IncI1-positive isolates that were evaluated for antimicrobial resistance, conjugal transfer ability and ability to inhibit the growth of an *E. coli* strain (24). Isolates selected belonged to serovars Heidelberg (*n* = 18, 42%), Typhimurium (*n* = 11, 26%), Newport (*n* = 6, 14%), Kentucky (*n* = 4, 9%) and Anatum, Dublin, Cerro, and Montevideo (*n* = 1, 2% each) (Table 1). Isolates originated from poultry (*n* = 17, 40%), cattle (*n* = 13, 30%), swine (*n* = 8, 19%) and human patients (*n* = 5, 12%) within the U.S. from 1999 to 2009 (16, 25, 26). *E. coli* J53 (27, 28) was used as a recipient strain for *E. coli* transconjugants generated in a previous study (24), but further characterized here. The following strains from ATCC or the FDA NCTR culture collection were used to assess their susceptibility to bacteriocins produced by wildtype *Salmonella* isolates and transconjugants: *Pseudomonas aeruginosa* ATCC 27853, *Enterococcus faecalis* ATCC 29212, *Staphylococcus aureus* ATCC 29213, *S. enterica* serovar Typhimurium ATCC 14028, *Klebsiella pneumoniae* N950, *Enterococcus cloacae* N1075, and the following *E. coli* strains ATCC 25922, N734 (*E. coli* O157:H7), 164, 524, 542, 550, 586, and 590 (29).

**Table 1:** Epidemiological information and WGS experimental results for the *Salmonella enterica* isolates.

							**IncI1 pMLST**						**cgMLST**
**Isolate**	**Serotype**	**Source**	**Year**	**State**	**GenBank Accession**	**PlasmidFinder**	***repI***	***ardA***	***trbA***	***sogS***	***pilL***	**ST**	**cgST**
67	Newport	Cattle	2002	IA	VCBN00000000	I1, A/C	5	4	5	4	2		15593
74	Newport	Cattle	2002	WA	VCBO00000000	I1, A/C	2	ND	5	4	3		16836
76	Newport	Chicken	2001	GA	VCBP00000000	I1, A/C	2	4	5	4	2		14763
89	Newport	Swine	2001	UT	VCIK00000000	I1, A/C	2	ND	5	4	3		18503
93	Newport	Swine	2002	KS	VCIL00000000	I1, A/C	1	4	13	2	1	26	18560
100	Newport	Turkey	2001	ND	VCIM00000000	I1	1	4	13	2	1	26	13903
111	Heidelberg	Cattle	2001	OH	VCIN00000000	I1, A/C, HI2	1	3	8	ND	10		43318
114	Heidelberg	Cattle	2002	IA	VCIO00000000	I1, A/C, HI2	1	3	8	ND	10		43318
115	Heidelberg	Cattle	2002	IN	VCIP00000000	I1, A/C, HI2	1	3	8	ND	10		43318
116	Heidelberg	Cattle	2002	IN	VCIQ00000000	I1, A/C, HI2	2	ND	5	4	2		43318
121	Heidelberg	Cattle	2002	N/A	VCSL00000000	I1, A/C	3	2	17	3	3		42871
142	Heidelberg	Swine	2002	IN	NPFC00000000	I1, A/C, HI2	1	4	13	2	1	26	18855
143	Heidelberg	Swine	2002	MN	NPEL00000000	I1, A/C, I2, HI2	1	4	13	2	1	26	18855
144	Heidelberg	Swine	2002	MN	NPEQ00000000	I1, A/C, HI2	3	3	5	4	3		17682
146	Heidelberg	Swine	2002	MN	NPEM00000000	I1, A/C, Col(BS512), I2, HI2, X4	1	2	3	9	2	266	42898
159	Heidelberg	Turkey	2002	NC	VCIR00000000	I1, HI2	1	4	3	4	1	12	108562
470	Typhimurium	Swine	1999	N/A	VCSK00000000	I1, Col156, Col440II, FIA, FIB, FIC, FII, HI1, X1, X4, p0111	1	4	5	4	1	25	16704
471	Typhimurium	Swine	1999	N/A	VCIT00000000	I1, ColpVC, FIB, FII	1	4	13	2	1	26	15908
482	Typhimurium	Turkey	1999	N/A	VCIU00000000	I1	1	1	3	9	3		98498
695	Heidelberg	Turkey	2000	MW*	VCIV00000000	I1, HI2	1	4	13	2	1	26	17326
706	Heidelberg	Turkey	2000	MW*	VDBX00000000	I1, Col(BS512), X1, X4	1	4	13	2	1	26	13882
715	Heidelberg	Turkey	2000	MW*	VCPT00000000	I1, X1	1	4	13	2	1	26	13882
849	Dublin	Cattle	2005	AZ	VCPU00000000	I1, A/C, FII, X1	1	38	16	9	2		168924
855	Typhimurium	Cattle	2006	WI	VCPV00000000	I1, FIB, FII	1	1	5	4	2		170278
856	Cerro	Cattle	2006	WI	VCPW00000000	I1	3	2	ND	4	3		46285
880	Montevideo	Cattle	2006	WI	VCIS00000000	I1	1	3	3	ND	ND		109916
891	Anatum	Cattle	2006	WI	VCPY00000000	I1	1	4	5	4	2	80	174486
990	Heidelberg	Human	2008	AR	VCQK00000000	I1, ColpVC, HI2	1	4	13	2	1	26	20340
991	Heidelberg	Human	2009	AR	NPEP00000000	I1, HI2	2	3	3	10	2	268	18855
1000	Heidelberg	Human	2009	NY	VCPZ00000000	I1, ColpVC, HI2	1	4	13	2	1	26	18855
1148	Heidelberg	Human	2007	WI	NPEO00000000	I1	1	4	13	2	1	26	4187
1163	Heidelberg	Human	2007	WI	VCQB00000000	I1, ColpVC	1	4	3	4	1	12	22762
N134	Typhimurium	Chicken Farm	unknown	WV	VCQG00000000	I1, ColpVC, FIB, FIC	1	4	3	4	1	12	9141
N136	Typhimurium	Chicken Farm	unknown	WV	VCQC00000000	I1, ColpVC, FIB, FIC	1	4	3	4	1	12	9141
N36	Typhimurium	Chicken	unknown	WV	NPER00000000	I1, ColpVC, FIB, FIC, X1	1	4	3	4	1	12	15967
N53	Typhimurium	Chicken	unknown	WV	VCQH00000000	I1, A/C, ColpVC	1	4	3	4	1	12	14597
N74	Typhimurium	Chicken	unknown	WV	VCQD00000000	I1, ColpVC, FIB, FIC, X1	1	4	3	4	1	12	4469
N82	Typhimurium	Chicken Farm	unknown	WV	VCQE00000000	I1, A/C	1	4	3	4	1	12	17652
N822	Kentucky	Chicken Farm	2008	AR	VCQI00000000	I1, X1	3	2	17	3	3		21561
N860	Kentucky	Chicken	2008	AR	VDBM00000000	I1, Col, ColpVC, X1	3	2	17	3	3		21561
N865	Kentucky	Chicken	2008	AR	VCQJ00000000	I1, X1	3	2	17	3	3		629
N89	Kentucky	Chicken Farm	unknown	WV	NPES00000000	I1, ColpVC, HI2, X1	1	4	3	4	1	12	8054
N97	Typhimurium	Chicken	unknown	WV	VCQF00000000	I1, A/C	1	4	3	4	1	12	22772

* MW, Midwestern State, not further defined; N/A, information not available.

For pMLST, “ND” indicated that the loci was not detected in the WGS contigs.

*Table demographic data adapted from Kaldhone et al. (24)*.

### Whole Genome Sequencing (WGS)

Strains 142, 143, 144, 146, 991, 1148, N36, and N89 were sequenced as part of previous studies (24); while the remaining 35 strains were sequenced as part of the current study. DNA was extracted using a DNeasy Blood and Tissue kit (Qiagen, Valencia, CA, USA) and DNA sequencing libraries were constructed using the Nextera XT DNA Library Preparation kits (Illumina, San Diego, CA, USA) sequencing reactions were carried out on an Illumina MiSeq instrument using 2 × 250 paired-end format. The sequencing data was processed as described previously (30, 31). Genome sequences from the 43 individual strains were evaluated using PlasmidFinder (32) to predict replicon/incompatibility (Inc.,) groups, ResFinder (33) for antimicrobial resistance gene detection, cgMLSTFinder (34) for core genome multilocus sequence typing (cgMLST), pMLST (32) for plasmid MLST (pMLST) and Pathosystems Resource Integration Center (PATRIC) (35). PATRIC analyses were carried out to identify the presence of predicted virulence factors, including those associated with bacteriocin production. Virulence factor data was extracted from PATRIC, transformed to binary data and imported into BioNumerics (Applied Maths, Austin, TX) for phylogenetic analysis using Dice coefficients and the generation of a dendrogram using unweighted pair group means with averages (UPGMA) methods. DNA sequence data are available at the accession numbers shown in Table 1.

### Biofilm Production Assay

The ability of *Salmonella* strains, recipient *E. coli* J53 and their respective transconjugants to produce biofilms was evaluated using the polystyrene microtiter plate method (36). Briefly, each strain was grown overnight in tryptic soy broth (TSB) at 37°C. The following day, the cells were diluted 1:100 in TSB and 200 μL of each bacterial suspension were inoculated into three wells of sterile 96-well flat-bottomed polystyrene plates. Negative control wells containing only TSB were also inoculated in triplicate. The plates were covered and incubated statically for 24 h at 37°C. After incubation, the growth medium was removed, and the wells were washed three times with 250 μL of phosphate buffered saline (PBS). The attached bacteria were then fixed with 200 μL of methanol (Sigma-Aldrich, St. Louis, MO) per well for 15 min, after which the plates were emptied and dried at room temperature. The plates were stained with 50 μL per well of crystal violet stain (0.41%; Fisher Diagnostics, Middletown, VA) for 5 min. The stain was removed, and the plate was washed under running tap water and subsequently air-dried. Next, 200 μL of 33% glacial acetic acid (Sigma-Aldrich) was added to the wells and the optical density (OD) of each well was measured at 550 nm in a SpectrMax Absorbance Reader (Molecular Devices, San Jose, CA). For analysis, the absorbance of the blank was subtracted from the wells to compensate for background absorbance. The experiments were repeated and the arithmetic mean value and standard deviations of the OD values for the six replicates was calculated. The results were further analyzed using Excel (Microsoft, Redmond, WA) using a t-test for significant difference (*p* < 0.05) in biofilm formation relative to the negative control and/or *E. coli* J53 for the transconjugants.

### Bacteriocin Inhibition Assay

The ability of *Salmonella* and transconjugant strains to produce colicins was evaluated by assessing growth inhibition of *E. coli* J53 (24) and the *P. aeruginosa, E. faecalis, S. aureus, S. enterica, K. pneumoniae, E. cloacae*, and the eight additional *E. coli* strains described above. Each of these isolates was grown overnight on tryptic soy agar with 5% sheep's blood and incubated overnight at 37°C. The following day bacterial growth was removed from the plates with a sterile swab and suspended in sterile demineralized water and the bacterial cell concentration adjusted to approximately 0.5 McFarland standard. The suspension was swabbed for confluence on blood agar plates and 5 μl of a *Salmonella* or *E. coli* transconjugant inoculum were prepared by suspending the equivalent of 5–6 bacterial colonies in 200 μl of sterile water, was spotted onto swabbed plate. The plates were incubated at 37°C for 16 to 18 hrs and then examined for growth inhibition of the bacterial lawn adjacent to the *Salmonella* or transconjugant growth. Inhibition was identified by a complete zone of growth inhibition around the *Salmonella* or transconjugant spots.

### Bacteriocin-Associated Gene Detection

For the wildtype *Salmonella* strains, bacteriocin-associated genes were identified from whole genome sequencing data using PATRIC and BLAST searching against the colicin genes. For the transconjugants, PCR reactions were carried out to detect the presence of 10 different bacteriocin-related genes *(cia, cib, colA, colD, colE1, colM, cvaA, cvaB, cvaC*, and *imm*). The primer sequences are listed in Table 2. Three to four colonies were collected from the plates and suspended in 200 μl of sterile water. The cells were lysed using a boiling method to prepare DNA template (37). PCR reactions included 2X Master Mix (Promega, Madison, WI), primer pairs (10 pmol), 2.5 μl of boiled template and sterile water to the final 25 μl volume. The genes were amplified with the following steps: denaturation at 95°C for 5 min, 30 cycles of denaturation at 95°C for 30 secs, annealing at either 52°C (*cvaA, cvaB*, and *cvaC*), 58°C (*cib* and *imm*) or 60°C (*cia, colA, colD, colE1*, and *colM*) for 30 secs, and extension at 72°C for 90 s, and a final extension at 72°C for 7 min. The resulting PCR products were separated on 2% E-gels (Invitrogen, Carlsbad, CA) and visualized under UV-light using a Gel-Doc XR system (Bio-Rad, Hercules, CA).

**Table 2:** Bacteriocin gene primers.

**Gene**	**Forward sequence**	**Reverse sequence**	**Annealing Temp (^**°**^C)**	**References**
*cia*(*col1a*)	CGAGATTTGCCGGTACGATAA	CGGTGACAGCCATCAGTAAA	60	(24)
*cib*	GGATGTGGAAGGTGACAAGAA	CACTCACAGCCGCCATAATA	58*	(24)
*colA*	TGCTCAGGATATGGCCGAATAAA	GCAGGAACTCCAAGAGATAAGG	60	This Study
*colD*	GGACTGCTGCTGGTGATATT	ACTACTGCTGGTAACGTGATTT	60	This Study
*colE1*	AACTGGCTGAAGCTGAAGAG	GCGATACCGGCATTACGATTA	60	This Study
*colM*	GTGGCATGTGTGAACTTTGTAG	CTGGTTCTTCACCCTGGAATAA	60	This Study
*cvaA*	CTATAGCCGCCGTGTTAATG	GACTCTCCAGAGTGGTTATCT	53	This Study
*cvaB*	GGCTGTTCGATCATCTTCTC	CCATCCTGGTTAGCTTAATACC	53	This Study
*cvaC*	CTGTTTCTGGTGGTGCTT	CCATGCTTCTGAAGGTATCC	53	This Study
*imm*	GGCAACCACAGGAACTGATA	GGATGGAAAGATAGCCAGGAAA	58*	(24)

* *Annealing temperature adjusted (58°C) from original reference (60°C)*.

### pMLST

For the wildtype *Salmonella* strains, pMLST profiles were identified from whole genome sequencing data. For the transconjugants, DNA template were prepared as previously described above for PCR and the *repI, ardA, trbA, sogS*, and *pilL* gene loci were amplified following the methods outlined previously (38). The resulting PCR products were separated on 2% E-gels, visualized under UV-light and the positive PCR reactions purified using the QIAquick PCR Purification Kit (Qiagen). The purified PCR products were sequenced at the University of Arkansas for Medical Sciences Sequencing Core Facility. The fasta files from WGS and individual allele-specific sequencing reads were submitted for identification of pMLST alleles and sequence types through the IncI1 pMLST database (https://pubmlst.org/plasmid/).

### Tissue Culture

Caco-2 cells were grown in Modified Eagle Medium (Corning, Manassas, VA) supplemented with 10% FBS, 1% of Pen/Strep/Amphotericin B (Sigma-Aldrich, St. Louis, MO), amino acids (Lonza, Walkersville, MD), and Glutamax (Thermo Fisher Scientific, Waltham, MA). Cells were grown in a 37°C incubator with 5% CO_2_ atmosphere. Prior to infection, *Salmonella* isolates were grown in LB broth overnight. The following day, the optical density of the cell suspension was measured with a spectrophotometer (Genesys 20, Thermo Fisher Scientific, Waltham, MA) at 600 nm and the predicted number of bacteria was calculated. Caco-2 cells were treated with trypsin and dispersed into 24-well culture plates and grown to confluence. The antibiotic-containing media was removed, the culture cells washed with sterile media and representative wells enumerated to determine the numbers of Caco-2 cells/well. Each experiment was conducted with three replicates and repeated for a total of six infections.

### Invasion Assay

Caco-2 cells were then infected with a 10 times greater number of *Salmonella* (i.e., multiplicity of infection 10:1). After infection, the cells were incubated at 37°C in 5% CO_2_ for 1 h. Gentamicin (200 μg/ml) was added to each well, and the cells were incubated for another hour. Cells were subsequently washed three times with PBS and then lysed with 0.1% 4°C Triton-X and suspensions were serially diluted followed by plating on LB agar plates. After overnight incubation at 37°C, the cells on the plates were enumerated to quantify the number of bacteria that invaded the Caco-2 cells (39).

### Persistence Assay

After an initial 1-h incubation, gentamicin (100 μg/ml) was added to each well and the plates were incubated for 48 h. After which, the cells were washed, lysed and plated as described for the invasion assay.

### Statistical Analyses

For each of the invasion and persistence experiments, statistical analyses were conducted using Microsoft Excel (ver. 2016, Redmond, WA). To normalize the impact of inoculum differences across the experiments, each bacterial cell count was divided by respective inoculum dose to obtain the relative invasion or persistence ratios. The average of the ratios of the six replicates was calculated along with the standard deviation for each set. Differences between the cell survival of invasion and persistence were evaluated to facilitate an understanding the contribution of the different plasmids using two-tailed paired *T*-test and *p* < 0.05 was considered as a significant difference between two groups compared.

## Results

**Figure 1:**
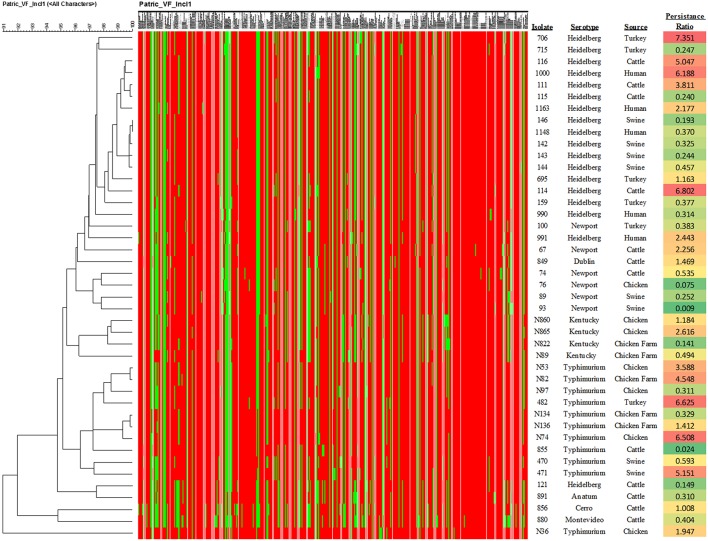
Results of the phylogenetic analysis of the virulence factor profiles. The heatmap indicate the presence (red) or absence (green) of specific virulence genes as shown in Supplemental Table 2. In addition, the calculation of the average ratio of persistence cell counts to the inoculum levels are shown in the right-hand column. The color shading of the values is based on the ratio, with green at the lower levels of persistence through red at the higher levels.

Forty-three IncI1 positive *S. enterica* isolates, representing eight different serovars were examined in the present study (Tables 1, 3). From these strains, 12 transconjugant containing IncI1 plasmids, but lacking other known plasmids were produced in recipient *E. coli* J53 and were further evaluated for the impact of IncI1 plasmids on bacterial physiology (Table 4). Each of the *Salmonella* isolates underwent WGS analyses and the sequencing assemblies were deposited in GenBank under the accession numbers shown in Table 1. The assembled sequencing contigs were submitted to the ResFinder program to identify predicted antimicrobial resistance genes (Supplemental Table 1). All isolates were positive for *aac*(6′)-Iaa, which is not associated with a resistance phenotype in *Salmonella* (40) and not included in the table. All but three isolates (856, 891, and N865), were positive for at least one additional antimicrobial resistance gene. The plasmid content of each strain was assessed using PlasmidFinder and the results presented in Table 1. All strains carried the IncI1 replicon and most carried additional identified replicon types, including IncA/C (42%, *n* = 18), IncHI2 (33%, *n* = 14), ColpVC (26%, *n* = 11), IncX1 (23%, *n* = 10), and IncFIB (16%, *n* = 7) (Table 1). In addition, the WGS data was analyzed using cgMLST and PATRIC to characterize the bacterial genomes. PATRIC identified putative virulence factors by comparison to their PATRIC-VF database that matched sequence annotations to the predicted *Salmonella* virulence factors. Supplemental Table 2 indicates the putative virulence factors predicted in the strains. The result of phylogenetic analysis based on the presence of the virulence factors is presented in Figure 1. Figure 1 also contains the data for the average ratio of bacterial persistence counts to the inoculum in Caco-2 cells for the respective isolates, which can serve as a phenotypic marker of potential virulence. The summary results of the cgMLST analyses are shown in Table 1, there were 34 different cgMLST types, with the largest groups being ST 18855 and 43318 (each 9%, *n* = 4). Supplemental Figure 1 shows a cladogram based on the cgMLST SNP analyses indicating a representation of the genetic relatedness of the strains.

**Table 3:** Results of the bacteriocin experiments for the *Salmonella enterica* isolates.

	**Bacteriocin inhibition assays**															**Bacteriocin-related gene detection**									
**Isolate**	***E. coli* J53**	***P. aeruginosa* 27853**	***E. fecalis* 29212**	***S. aureus* 29213**	***S*. Typhimurium 14028**	***K. pneumoniae***	***E. cloacae***	***E. coli* 25922**	***E. coli* O157:H7**	***E. coli* 164**	***E. coli* 524**	***E. coli* 542**	***E. coli* 550**	***E. coli* 586**	***E. coli* 590**	***cirA***	***omr***	***clp***	***cia***	***cib***	***cvpA***	***cvaC***	***colE***	***Imm* (Q2VNY0)**	***Imm* (V1L9U1)**
67	+	–	–	–	–	–	–	–	–	–	–	–	–	–	–	+	+	–	–	+	–	–	–	–	–
74	+	–	–	–	–	–	–	–	–	–	–	–	–	–	–	+	+	–	–	+	+	–	–	+	–
76	+	–	–	–	–	–	–	–	–	–	–	–	–	–	–	+	+	–	+	–	+	–	–	–	–
89	+	–	–	–	–	–	–	–	–	–	–	–	–	–	–	+	+	–	+	+	+	–	–	+	–
93	+	–	–	–	–	–	–	–	–	–	–	–	–	–	–	+	+	–	–	+	+	–	–	+	–
100	–	–	–	–	–	–	–	–	–	–	–	–	–	–	–	+	+	–	–	–	+	–	–	–	–
111	–	–	–	–	–	–	–	–	–	–	–	–	–	–	–	+	+	–	–	–	+	–	–	–	–
114	–	–	–	–	–	–	–	–	–	–	–	–	–	–	–	+	+	–	–	–	+	–	–	–	–
115	–	–	–	–	–	–	–	–	–	–	–	–	–	–	–	+	+	–	–	–	+	–	–	–	–
116	–	–	–	–	–	–	–	–	–	–	–	–	–	–	–	+	+	–	–	–	+	–	–	–	–
121	–	–	–	–	–	–	–	–	–	–	–	–	–	–	–	+	+	–	–	–	+	–	–	–	–
142	+	–	–	–	–	–	–	–	–	–	–	–	–	–	–	+	+	–	–	+	+	–	–	+	–
143	+	–	–	–	–	–	–	–	–	–	–	–	–	–	–	+	+	–	–	+	+	–	–	+	–
144	–	–	–	–	–	–	–	–	–	–	–	–	–	–	–	+	+	–	–	–	+	–	–	–	–
146	+	–	–	–	–	–	–	–	–	–	–	–	–	–	–	+	+	–	–	+	+	–	–	+	–
159	+	–	–	–	–	–	–	–	–	–	–	–	–	–	–	+	+	–	–	+	+	–	–	+	–
470	+	–	–	–	–	–	–	–	–	–	–	–	–	–	–	+	+	+	–	+	+	–	+	+	–
471	+	–	–	–	–	–	–	–	–	–	–	–	–	–	–	+	+	–	–	+	+	–	–	+	–
482	+	–	–	–	–	–	–	–	–	–	–	–	–	–	–	+	+	–	–	+	+	–	–	+	–
695	+	–	–	–	–	–	–	–	–	–	–	–	–	–	–	+	+	–	–	+	+	–	–	+	–
706	+	–	–	–	–	–	–	–	–	–	–	–	–	–	–	+	+	–	–	+	+	–	–	+	–
715	+	–	–	–	–	–	–	–	–	–	–	–	–	–	–	+	+	–	–	+	+	–	–	+	–
849	–	–	–	–	–	–	–	–	–	–	–	–	–	–	–	+	+	–	–	–	+	–	–	–	–
855	+	–	–	–	–	–	–	–	–	–	–	–	–	–	–	+	+	–	–	+	+	–	–	+	–
856	–	–	–	–	–	–	–	–	–	–	–	–	–	–	–	+	+	–	–	–	+	–	–	–	–
880	–	–	–	–	–	–	–	–	–	–	–	–	–	–	–	+	+	–	–	+	+	–	–	–	–
891	–	–	–	–	–	–	–	–	–	–	–	–	–	–	–	+	+	–	–	–	+	–	–	–	–
990	+	–	–	–	–	–	–	–	–	–	–	–	–	–	–	+	+	–	–	+	+	–	–	+	–
991	–	–	–	–	–	–	–	–	–	–	–	–	–	–	–	+	+	–	–	+	+	+	–	–	–
1000	+	–	–	–	–	–	–	–	–	–	–	–	–	–	–	+	+	–	–	+	+	–	–	+	–
1148	+	–	–	–	–	–	–	–	–	–	–	–	–	–	–	+	+	–	–	+	+	–	–	+	–
1163	+	–	–	–	–	–	–	–	–	–	–	–	–	–	–	+	+	–	–	+	+	–	–	+	–
N134	+	–	–	–	–	–	–	–	+	–	–	+	–	–	+	+	+	–	–	+	+	+	–	+	–
N136	+	–	–	–	–	–	–	–	+	–	–	+	–	–	+	+	+	–	–	+	+	+	–	+	–
N36	+	–	–	–	–	–	–	–	–	–	–	–	–	–	–	+	+	–	–	+	+	–	–	+	–
N53	+	–	–	–	–	–	–	–	–	–	–	–	–	–	–	+	+	–	–	+	+	–	–	+	–
N74	+	–	–	–	–	–	–	–	–	–	–	–	–	–	–	+	+	–	–	+	+	–	–	+	–
N82	+	–	–	–	–	–	–	–	–	–	–	–	–	–	–	+	+	–	–	+	+	–	–	+	–
N822	–	–	–	–	–	–	–	–	–	–	–	–	–	–	–	+	+	–	–	+	+	–	–	–	+
N860	–	–	–	–	–	–	–	–	–	–	–	–	–	–	–	+	+	–	–	+	+	–	–	–	+
N865	–	–	–	–	–	–	–	–	–	–	–	–	–	–	–	+	+	–	–	+	+	–	–	–	+
N89	+	–	–	–	–	–	–	–	–	–	–	–	–	–	–	+	+	–	–	+	+	–	–	+	+
N97	+	–	–	–	–	–	–	–	–	–	–	–	–	–	–	+	+	–	–	+	+	–	–	+	–

For the Bacteriocin Inhibition Assay: “+” indicates complete inhibition and “–” indicates no apparent inhibition.

*For the Bacteriocin Gene Detection: “+” indicates the presence and “–” indicates a absence of the gene in WGS data*.

**Table 4:** IncI1-positive transconjugants and the results of the pMLST and bacteriocin studies.

	**pMLSTAllele and ST**						**Bacteriocin inhibition assays**															**Bacteriocin gene detection**									
**Transconjugant**	***repI***	***ardA***	***trbA***	***sogS***	***pilL***	**ST**	***E. coli* J53**	***P. aeruginosa* 27853**	***E. fecalis* 29212**	***S. aureus* 29213**	***S*. Typhimurium 14028**	***K. pneumoniae***	***E. cloacae***	***E. coli* 25922**	***E. coli* O157:H7**	***E. coli* 164**	***E. coli* 524**	***E. coli* 542**	***E. coli* 550**	***E. coli* 586**	***E. coli* 590**	***cia***	***cib***	***colA***	***colD***	***colE1***	***colM***	***cvaA***	***cvaB***	***cvaC***	***Imm***
X100	1	4	13	2	1	26	–	–	–	–	–	–	–	–	–	–	–	–	–	–	–	–	–	–	–	–	–	–	–	–	–
X116	8	4*	5	4	2	Unique	–	–	–	–	–	–	–	–	–	–	–	–	–	–	–	–	–	–	–	–	–	–	–	–	–
X121	3	2	17	3	3	Unique	–	–	–	–	–	–	–	–	–	–	–	–	–	–	–	–	–	–	–	–	–	–	–	–	–
X159	1	4	3	4	1	12	+	–	–	–	–	–	–	–	–	–	–	–	–	–	–	–	+	–	–	–	–	–	–	–	+
X471	1	4	13	2	1	26	+	–	–	–	–	–	–	–	–	–	–	–	–	–	–	–	+	–	–	–	–	–	–	–	+
X482	1	1	3	9	3	Unique	+	–	–	–	–	–	–	–	–	–	–	–	–	–	–	–	+	–	–	–	–	–	–	–	+
X695	1	4	13	2	1	26	+	–	–	–	–	–	–	–	–	–	–	–	–	–	–	–	+	–	–	–	–	–	–	–	+
X706	1	4	13	2	1	26	+	–	–	–	–	–	–	–	–	–	–	–	–	–	–	–	+	–	–	–	–	–	–	–	+
X715	1	4	13	2	1	26	+	–	–	–	–	–	–	–	–	–	–	–	–	–	–	–	+	–	–	–	–	–	–	–	+
X891	1	4	5	4	2	80	–	–	–	–	–	–	–	–	–	–	–	–	–	–	–	–	–	–	–	–	–	–	–	–	–
X1000	1	4	13	2	1	26	+	–	–	–	–	–	–	–	–	–	–	–	–	–	–	–	+	–	–	–	–	–	–	–	+
XN53	1	4	3	4	1	12	+	–	–	–	–	–	–	–	–	–	–	–	–	–	–	–	+	–	–	–	–	–	–	–	+

For the Colicin Inhibition Assay: “+” indicates complete inhibition and “–” indicates no apparent inhibition.

For the Colicin Gene Detection: “+” indicates a positive PCR reaction and “–” indicates a negative PCR reaction.

*Non-identical allele:closest match.

The results of the IncI1 pMLST experiments of the *Salmonella* isolates identified 18 different allele profiles (Table 1). The most common STs identified were 26 (26%, *n* = 11), 12 (23%, *n* = 10), and an undefined ST with the allele profile (3, 2, 17, 3, 3; Table 1, 9%, *n* = 4). For seven of the isolates not all five alleles were detected; however, three (7%) and two (5%) of these isolates had shared overall profiles (Table 1). Among the 12 IncI1 transconjugants evaluate, there were six different allele profiles, including three that did not have a previously identified STs. The most common STs were 26 (50%, *n* = 6) and 12 (17%, *n* = 2) (Table 4).

The results of the bacteriocin inhibition assays and PCR results for the *Salmonella* isolates are shown in Table 1. None of the IncI1-positive *Salmonella* isolates inhibited the non-*E. coli* strains, including *P. aeruginosa, E. faecalis, S. aureus, S. enterica, K. pneumoniae*, and *E. cloacae*. As noted previously (24), the majority of isolates (65%, 28/43) were able to inhibit the growth of *E. coli* 53 (27). For the other seven *E. coli* strains tested, the results were varied, none of the isolates inhibited *E. coli* ATCC 25922 and veterinary clinical strains 164, 524, 550, and 586. *E. coli* O157:H7 isolate 734, and veterinary *E. coli* isolates 542 and 590 were inhibited by *Salmonella* isolates N134 and N136 (Table 3). The detection of the bacteriocin-associated genes in the *Salmonella* isolates are shown in Table 3. All of the isolates were positive for the colicin receptor genes *cirA* and *omr* and the colicin v-associated gene *cvpA*, with the exception of isolate 67 for *cvpA*. Among the predicted bacteriocin-encoding gene families, colicin lysis protein (*clp*: *n* = 1, 2%), channel-forming colicins (*cib*: *n* = 32, 74% and *cia*: *n* = 2, 5%), colicin production (*cvpA*: *n* = 42, 98%*, cvaC*: *n* = 3, 7%*, colE*: *n* = 1, 2%) were detected. Two different colicin immunity proteins were predicted among the strains (NCBI protein ID: Q2VNY0: *n* = 26, 60%) and (protein ID: V1L9U1: *n* = 4, 9%). All strains that were able to inhibit *E. coli* J53 growth carried either *cib* (*n* = 27, 96%) and/or *cia* (*n* = 2, 7%), and with the exception of isolates 67 and 76 carried the colicin b immunity (*imm*) gene (Table 3). Isolate 76 was negative for *cib*, but positive for *cia. Five* strains were stains were positive for *cib* but did not inhibit *E. coli* J53, each of these lacked the colicin immunity gene (Q2VNY0). Isolates N134 and N136, which inhibited the greatest number of *E. coli* were positive for *cib, cvpA, cvaC*, and *imm*. Isolate 991, was positive for *cib, cvpA*, and *cvaC*, but negative for *imm* and subsequently there was no observed inhibition of any of the bacterial strains.

Of the 12 IncI1 transconjugants, eight (67%) were able to inhibit the growth of *E. coli* J53, but did not inhibit the growth of any of the other strains tested, including all other *E. coli* (Table 4). All eight of the transconjugants that inhibited *E. coli* J53 carried the *cib* and *imm* genes, while those that did not inhibit growth were negative for each of the bacteriocin genes tested (Table 4). Each of the strains that inhibited the growth of *E. coli* J53 were either ST26 (*n* = 5), ST12 (*n* = 2) or an unclassified ST (*n* = 1).

The biofilm experiments demonstrated that there was variability of the wild-type strains to form biofilms. Fifteen of the 43 (35%) isolates had significantly increased absorbance versus the negative control, indicating an ability to form biofilms. All 12 transconjugants appeared to have biofilm formation ability; however only two (17%) reached the level of significance. The recipient strain *E. coli* J53 also had non-significantly higher absorbance (*p* = 0.11). When the biofilm forming ability of the transconjugants was compared to *E. coli* J53, none had significantly greater biofilm ability than J53 alone, indicating that the potential contribution of the plasmid(s) to biofilm formation is minimal.

**Figure 2:**
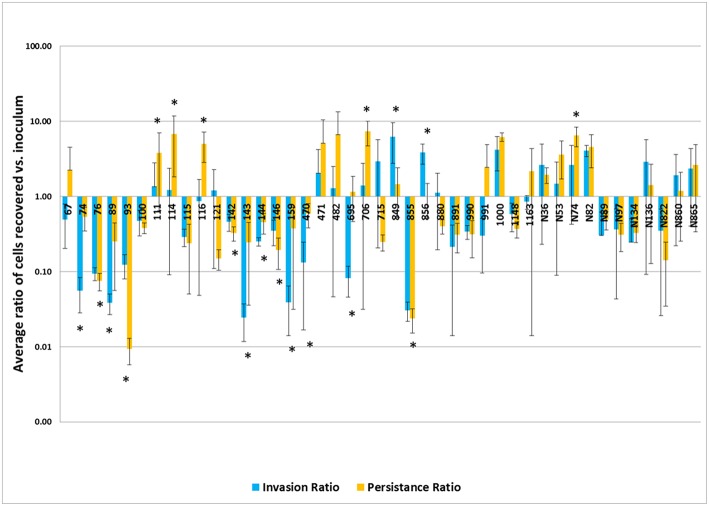
Results of the invasion and persistence assays. The invasion bars (light blue) indicate the average of ratios of cells recovered vs. inoculum detected after a 1 h invasion period and persistence bars (orange) indicate the average of ratios of cells recovered vs. inoculum detected following a 48 h incubation period. Each set of experiments was done in triplicate and repeated. The error bars indicate the standard deviation across the 6 counts for each isolate. Isolate numbers are noted on X-axis, while average of ratios of cells recovered vs. inoculum detected are expressed along Y-axis. *Indicates statistically significant difference between invasion and persistent averages of ratios for given isolate (*p* < 0.05).

The results of the invasion and persistence assays for the *Salmonella* isolates are displayed in Figure 2. All isolates tested could invade Caco-2 cells. Twelve of the forty-three (28%) isolates demonstrated significantly higher bacterial cell counts after 48 hrs of infection compared to their initial inoculum levels, which is indicative of intracellular growth. An additional 12 isolates (26%) exhibited increased cell numbers after 48 h that did not reach statistical significance. Seven of the isolates (16%) examined, yielded persistent cell populations significantly lower than those observed in the invasion assays (*p* < 0.05). An additional 12 (28%) showed decreased persistence that did not reach statistical significance.

When the ratio of persistence to the initial inoculum were examined based on serotype (Figure 1), the isolates from *S*. Newport demonstrated a significantly lower survival in host cells than those of serovars Typhimurium (*p* = 0.00015) and Heidelberg (*p* = 0.0036), while *S*. Kentucky strains had significantly lower persistence than those of *S*. Typhimurium (*p* = 0.0098), but not *S*. Heidelberg (*p* = 0.089). Similarly, when the sources of the isolates were compared, isolates collected from swine showed a significantly lower persistence in Caco-2 cells than those isolated from chickens (*p* = 0.007), turkeys (*p* = 0.015), and human patients (*p* = 0.017). There were no significant differences between isolates containing the three most common pMLST types (noted above) and increased persistence within the Caco-2 cells.

## Discussion

*Salmonella enterica* can infect several different hosts, with their transmission being primarily via the fecal-oral route (5). Several *Salmonella* strains have the ability to form biofilms that allow groups of cells to be more resilient to killing and persist in the environment, potentially facilitating contamination of food products, animal production environments and within host organisms (36). After ingestion, *Salmonella* enters the intestinal tract and can cause gastroenteritis, the most common illness caused by *Salmonella* infection. To cause infection, *Salmonella* must compete with intestinal bacteria and colonize the intestinal epithelia. The production of bacteriocins can provide a competitive advantage for the *Salmonella* against members of the microbiota, such as *E. coli* (41, 42). Intestinal epithelial cells provide *Salmonella* a niche for colonization in their host (43). A type 3 secretion system (T3SS) encoded by *Salmonella* pathogenicity island 1 (SPI-1) facilitates the uptake of the *Salmonella* into the host cells (44). After entering epithelial cells *Salmonella* become engulfed into *Salmonella*-containing vacuoles (SCV). Within the SCV, *Salmonella* can move toward the basal side of the epithelium where they can be taken up by macrophages or dendritic cells (45). Through these immune cells, *Salmonella* can enter the lymphatic and systemic circulation leading to systemic infection (11). Therefore, intestinal epithelial cells play an important role in the pathogenesis of *Salmonella* infection. The Caco-2 cell line is derived from human intestinal epithelial cells and has been commonly used to study *Salmonella* pathogenesis (39, 43, 46). Caco-2 cells in combination with the WGS analyses were used in this study to gain a better understanding of IncI1 plasmid-positive *Salmonella* virulence, using invasion, persistence and intracellular proliferation in intestinal epithelial cells as the model system.

In the virulence assay, we observed that persistent cell populations after 48 hrs of infection were higher than the invasion cell counts in many of the isolates examined (Figure 2). All of the isolates in the study contained the genes encoding the SPI-1 T3SS apparatus (Supplemental Table 2), which likely facilitated the uptake of the *Salmonella* into the host cells (44). Among the different serovars tested, isolates from serovar Typhimurium appeared to have increased persistence capacity by showing higher average persistent cell counts, in general. *Salmonella* Typhimurium is known to be able to persist in the SCV, as well as the host cell cytosol (47, 48). Survival in the SCV is aided by a second T3SS encoded on SPI-2, whose effectors are important for intracellular survival. The presence of the SPI-2 encoded genes were identified in the strains and can contribute to the survival and proliferation within the host cells (49). Differences in the SPI-2 regulation and expression could give *S*. Typhimurium an advantage for persistence within intestinal epithelial cells, potentially allowing for more efficient intracellular multiplication than other serovars, however their exact contributions need to be further quantified. While statistical significance was observed, these numbers need to be evaluated in the context that there were uneven distributions of isolates from different sources and serotypes, with *S*. Newport and swine being on the lower end of representation, thus the impact of each isolate examined could disproportionately alter the overall results.

To attempt to get a better handle on the potential specific contribution that the IncI1 plasmids may have had on persistence and other characteristics examined, we used pMLST to subtype the IncI1 plasmids in the *Salmonella* isolates and the transconjugants. If there were particular IncI1 plasmid types that were associated with the more persistent phenotypes, pMLST might help discern these differences. Based on the results of the Caco-2 tissue culture experiments and corresponding results of pMLST, there did not appear to a connection between plasmid type and persistence in the epithelial cells. Of the strains that had significantly increased and decreased persistence or intracellular multiplication compared to the initial inoculum, there were overlaps in plasmid types, for example, isolates 706 and 93 had the same allele profiles (ST 26), yet opposite persistence profiles, with strain 706 having the highest ratio of cell numbers to inoculum at 48 hrs and 93 the lowest (Figure 1). Ideally, these invasion and persistence experiments would have been completed in the transconjugants or in plasmid-cured *Salmonella*; however, attempts to cure the plasmids or transfer the plasmids into a well-characterized *Salmonella* strain to conduct the invasion and persistence experiments were unsuccessful in the present study. Also, the *E. coli* transconjugants generated were not ideal for the comparative assessment of invasion and persistence because they lack the T3SS important for entry into intestinal epithelial cells. Our ongoing efforts in the lab are focused on optimization of methods for plasmid curing and transfer of plasmids into *Salmonella* recipients which facilitate further genetic characterization of these and other plasmids. Because isolates with similar plasmid types spanned the spectrum of persistence, from significantly reduced persistence to significantly increased cell numbers, the results indicate that the IncI1 plasmids may not be major contributors to persistence in *Salmonella*.

**Figure 3:**
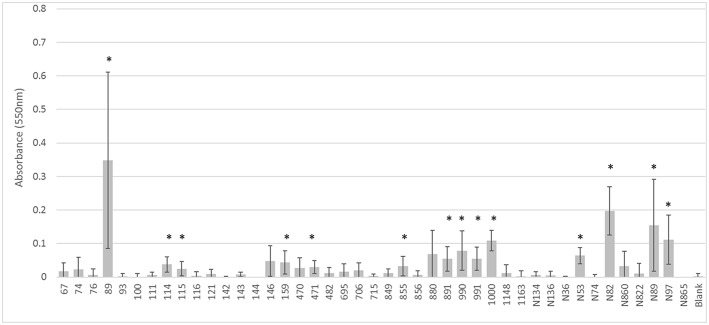
Results of the biofilm assays for the wildtype strains. The bars indicate the average absorbance at 550 nm of the stained cells attached to the assay plates. Each set of experiments involved 3 replicates and the experiments were repeated. The error bars indicate the standard deviation across the 6 absorbance readings for each isolate. Isolate numbers are noted on X-axis, while average absorbance is expressed along Y-axis. *Indicates statistically significant difference between the isolate and the non-inoculated blank (*p* < 0.05).

**Figure 4:**
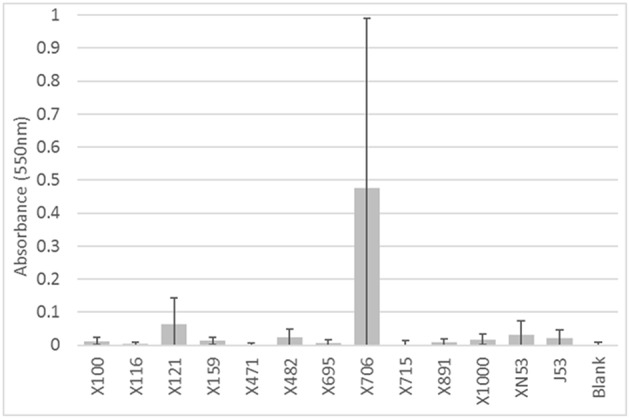
Results of the biofilm assays for the transconjugants and *E. coli* J53 recipient. The bars indicate the average absorbance at 550 nm of the stained cells attached to the assay plates. Each set of experiments involved 3 replicates and the experiments were repeated. The error bars indicate the standard deviation across the 6 absorbance readings for each isolate. Isolate numbers are noted on X-axis, while average absorbance is expressed along Y-axis. None of the transconjugants had a significantly higher (*p* < 0.05) absorbance than the recipient strain *E. coli* J53.

Likewise, the IncI1 plasmids did not play a significant role in the ability to form biofilms. While some of the strains formed biofilms, there was no consistent trend across the IncI1-positive isolates (Figure 3). Over half of the strains tested did not have significantly more biofilm formation compared to the blank wells which had no cells. Furthermore, in the experiments with transconjugants, the addition of the IncI1 plasmids to the recipient strain did not significantly increase the biofilm producing ability of *E. coli* J53 (Figure 4).

In addition to the biofilm and persistence studies, this study examined the production of bacteriocins and their potential ability to inhibit multiple species including enteric organisms such as *S. enterica, K. pneumoniae, E. cloacae*, and *E. coli*. Our earlier work indicated that several of the *Salmonella* isolates and transconjugants generated from these strains were able to inhibit *E. coli* J53 (24), thus in the current study we examined multiple *E. coli* isolates, the other enteric organisms noted above, and potential pathogens such as *P. aeruginosa, E. faecalis*, and *S. aureus* to further explore the potential spectrum of activity. When bacteriocin-associated genes were evaluated, some isolates were positive for multiple bacteriocin genes. Even with the multiple genes, the spectrum of activity for most isolates was narrow, only inhibiting a limited number of *E. coli*. Two isolates N134 and N136 were able to inhibit four different *E. coli*, including an *E. coli* O157:H7 strain. These two isolates contained multiple bacteriocin-associated genes, such as *cib*, which is commonly associated with IncI1 plasmids (50) and *cvaC* that is typically associated with IncFIB plasmids (51). Both isolates were positive for both IncI1 and IncFIB plasmids, however neither plasmid appeared to be conjugative for either isolate so were not able to evaluate their impact in the transconjugants.

The development of the transconjugants in *E. coli* generated using the well-characterized *E. coli* J53 strain (27, 28), helped to determine the impact of the IncI1 plasmids on bacterial inhibition. The majority (67%) of transconjugants that carried IncI1 plasmid were able to inhibit the growth of *E. coli* J53. All of these transconjugants received the colicin immunity gene, *imm* and *cib* bacteriocin gene. The IncI1 plasmids from each of these strains fell into ST 26, 12 or a unique profile (isolate 482). One *Salmonella* isolate (100) with ST 26 IncI1 plasmid lacked *cib* or *imm* and was not able to inhibit the grow of any strains tested, which was contrary to all other ST 26 plasmid-positive isolates. Taken together, these results demonstrate the importance of specific IncI1 plasmids for the dissemination of bacteriocins that may provide a selective advantage in an environment containing certain *E. coli* populations.

IncI1 plasmids may persist in *Salmonella* populations due to their contribution to antimicrobial resistance and bacteriocin production but at the same time appear to have minimal metabolic costs on the host strains. Johnson et al. (52) showed that the acquisition of IncI1 plasmids did not significantly add to the fitness cost of a host bacterium and in some cases led to a negative fitness cost (i.e., increased fitness) to the strains that acquired them. In strains carrying IncA/C plasmids, the fitness cost of also acquiring an IncI1 plasmid was no greater than carrying the IncA/C plasmid alone or in some cases a negative cost (52). This may help explain why many of the isolates with the greatest ability to multiply and persist in the epithelial cells (i.e., highest persistence/invasion ratios) carried multiple large plasmids, including IncA/C, IncHI2, IncFIB, and IncX (Figure 2 and Table 1).

Additionally, there is evidence that intestinal inflammation and iron limitation may impact bacteriocin function due the expression of the enterobactin siderophore (iron chelator) receptors on the bacterial cell surface, which can also serve as a receptor for colicin 1b (encoded by *cib*) (41, 53). Results from the PATRIC-VF analyses show that each of the strains have the siderophore receptors. With the increased expression of the colicin receptors by commensal *E. coli*, it may allow the *Salmonella* to impair their competitors for nutrients and colonization that facilitates uptake into the intestinal epithelial cells (42). The presence of plasmid-associated genes within the host may also impact the susceptibility to bacteriocins, expression of plasmid-associated siderophores, salmochelin, and aerobactin, by *E. coli* have been shown to limit the impact of *Salmonella* in the gastrointestinal tract (54). Interestingly, the two isolates that inhibited the greatest number of *E. coli* (N134 and N136) carried an IncFIB plasmid in addition to IncI1 and the veterinary *E. coli* strains that they inhibited (542 and 590) lacked the aerobactin operon, while the four (164, 524, 550, and 586) not inhibited were positive for the aerobactin operon (29), which is commonly carried by IncFIB plasmids (39). The reason for this dichotomy could either be due to *E. coli* carrying an IncFIB-encoded immunity protein or having the aerobactin machinery to acquire iron without needing to highly express the enterobactin receptor. Thus, there is a continued need to be decipher the biology of plasmids to help understand factors that contribute to the public health challenges faced by consumers.

## Conclusions

*Salmonella* are important foodborne pathogens in humans and are major concerns for food animal production. Increased virulence of *Salmonella* and the capability to disseminate virulence determinants rapidly on plasmids, make them a concern for public health. This study was undertaken to characterize IncI1 plasmid carrying *Salmonella* isolates and identify potential contributions of IncI1 plasmids to virulence. The WGS analyses indicated that the IncI1 plasmids are spread across a generically diverse range of *Salmonella* including multiple serotypes, cgMLST profiles and having diverse virulence gene profiles. Additionally, there was diversity among the IncI1 plasmids evaluated in the study as evidenced by the multiple pMLST subtypes among the *Salmonella* isolates evaluated in the study. While these plasmids were not apparent significant contributors to increased biofilm formation or persistence in intestinal epithelial cells, the study does indicate a role of specific IncI1 plasmids to inhibit the growth of other bacteria and potentially contribute to virulence. Bacteriocins can provide a colonization advantage to *Salmonella* and subsequently facilitate invasion of the intestinal epithelial cells. The study demonstrated that *Salmonella* containing IncI1 plasmids frequently carry multiple antimicrobial resistance genes and can persist and often multiply with human intestinal epithelial cells. Overall, the study shows that it is important to begin to decipher areas of virulence where IncI1 plasmids likely contribute, while at the same time describing other areas where they do not. These results will allow us to hone in on specific plasmid-encoded factors that impact bacterial pathophysiology and human health.

## Data Availability

The datasets generated for this study can be found in GenBank, Accession numbers are listed in Table 1.

## Author Contributions

SR and SF secured the funds for the project. SF, SR, PK, NA, and JH designed the studies. PK, AC, NA, JD, and BK conducted the laboratory studies. SF, PK, SR, NA, YS, and JH conducted the data analyses. PK, AC, SF, and SR wrote the final manuscript. All contributed to the editing and refinement of the finished manuscript.

### Conflict of Interest Statement

The authors declare that the research was conducted in the absence of any commercial or financial relationships that could be construed as a potential conflict of interest.

## Supplementary Material

Supplemental Table 1 Antimicrobial resistance genes detected by WGS analyses.

Supplemental Table 2 Virulence-associated genes detected from WGS data analyzed using the PATRIC-VF database.

Supplemental Figure 1 Cladogram of SNP analyses of WGS data. Isolate numbers in red indicate strains that had significantly greater persistence relative to invasion numbers and those in blue have significantly lower persistence numbers.
