# Supplementary figures and images for: Evaluation of Incompatibility Group I1 (IncI1) Plasmid-Containing Salmonella enterica and Assessment of the Plasmids in Bacteriocin Production and Biofilm Development
Source: Front Vet Sci. 2019 Sep 6;6:298. doi: 10.3389/fvets.2019.00298 (PMC6743044; doi:10.3389/fvets.2019.00298)

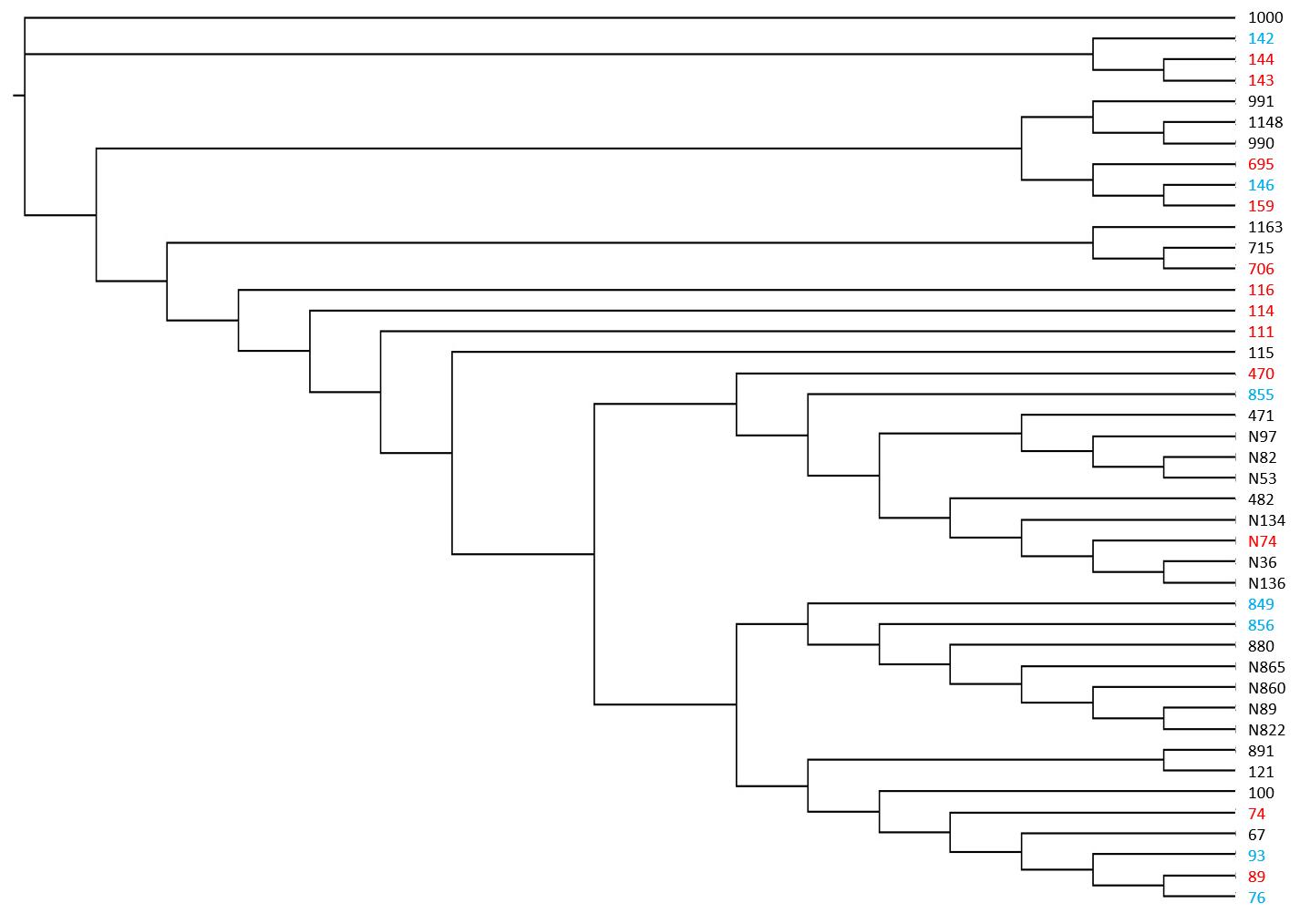

Supplement: Supplemental Figure 1 — Cladogram of SNP analyses of WGS data. Isolate numbers in red indicate strains that had significantly greater persistence relative to invasion numbers and those in blue have significantly lower persistence numbers. [file Image_1.TIF]
